# Cloning and high temperature tolerance analysis of the thermal response related gene *KcRCB* in *Karelinia caspia* (Pall.) Less

**DOI:** 10.3389/fpls.2025.1641916

**Published:** 2025-09-17

**Authors:** Wenlong Li, Wanyi Huang, Yanhai Zhao, Guoqiang Li, Shuguang Li, Yanqin Wang

**Affiliations:** Xinjiang Production and Construction Corps Key Laboratory of Protection and Utilization of Biological Resources in Tarim Basin, College of Life Science and Technology, Tarim University, Alar, Xinjiang, China

**Keywords:** *Karelinia caspia*, KcRCB, high temperature, expression analysis, functional exploration

## Abstract

**Introduction:**

The REGULATOR OF CHLOROPLAST BIOGENESIS (RCB) is a novel protein component in plant temperature signaling that functions by synergizing with HEMERA (HMR) to initiate thermomorphogenesis by stabilizing PHYTOCHROME INTERACTING TRANSCRIPTION FACTOR 4 (PIF4) during the day.

**Methods:**

In this study, we successfully cloned the heat-responsive gene KcRCB from Karelinia caspia, a desert-adapted plant species. KcRCB transcript levels were significantly elevated when the plants were exposed to high temperatures. Furthermore, KcRCB demonstrated differential expression in the Karelinia caspia roots, stems, and leaves, with optimal expression in the leaves. Subsequently, KcRCB transgene was overexpressed in Arabidopsis thaliana and cotton plants to characterize its thermomorphogenesis effects. In comparison with the wild-type Arabidopsis thaliana plants, KcRCB-overexpressing Arabidopsis thaliana plants exhibited reduced incidence of leaf damage and enhanced capacity to withstand elevated temperatures. KcRCB-overexpressing cotton plants subjected to elevated temperatures also exhibited reduced leaf damage.

**Results and Discussion:**

Physiological assays demonstrated that KcRCB expression enhances plant resilience to high-temperature stress by maintaining cell membrane stability and reducing the accumulation of reactive oxygen species (ROS). Moreover, we observed increased stomatal density and opening in the leaves of the KcRCB overexpressing lines compared to the control group when exposed to high temperatures. Subcellular localization experiments showed that KcRCB was localized to the stomatal guard cell membranes. This suggested that KcRCB protects plant cells from high-temperature-related damage by regulating stomatal openness, increasing the transpiration rate, and improving the efficiency of heat dissipation, thereby. These findings enhance the understanding of the mechanisms underlying high-temperature tolerance in the desert plant species. Specifically, this study expands our understanding regarding the biological roles of KcRCB and the molecular regulatory networks underlying heat stress responses in Karelinia caspia.

## 1 Introduction

In recent years, global warming has become a serious problem and is expected to significantly affect crop yields by 2040 (Challinor et al., 2014). Higher temperatures can significantly impact crop growth (Zhao et al., 2017), as well as plant phenology, distribution, and diversity, thereby significantly reducing crop productivity (Arnold et al., 2019; Rathore et al., 2020; Sadok et al., 2020). Plant photosynthesis is irreversibly damaged at temperatures beyond the critical threshold for plant growth (Bernacchi et al., 2025). Plants have an inherent ability to perceive and respond to environmental fluctuations and stresses (Lamers et al., 2020). Plants respond and adapt to rising temperatures by undergoing morphological changes through a process known as thermomorphogenesis (Franklin et al., 2011; Quint et al., 2016). The mechanisms underlying thermomorphogenesis and identification of heat responsive genes are current hotspot areas in plant research. *Arabidopsis thaliana* is the commonly used plant for studying thermal morphogenesis. It exhibits optimal growth at 22°C, but temperatures above 29°C significantly affect its growth, distort its structure and morphology, reduce seed yield, and cause significant temperature-related morphological adjustments (Gil and Park, 2018). The specific high temperature-related characteristics include elongation of hypocotyls, petioles, and stem, and leaf drooping (Casal and Balasubramanian, 2019). In terrestrial eudicots, elongation of hypocotyls and petioles represent significant thermal morphological responses of seedlings. For example, sustained warm temperatures cause elongation of hypocotyls and petioles in *Arabidopsis thaliana* (Blázquez et al., 2003; Crawford et al., 2012). Enhanced blade evaporative cooling is an adaptation to alleviate high temperatures (Blázquez et al., 2003). Furthermore, higher temperatures also lead to an increased ratio between stomata and epidermal cells, as well as narrower and longer taproots. Moreover, continuous higher temperatures will reduce the stomatal index (Legris et al., 2017; Pérez-Bueno et al., 2022).

PIF4 plays a key regulatory role in thermomorphogenesis when *Arabidopsis thaliana* plants are exposed to elevated temperatures. PIF4 directly regulates the expression of auxin biosynthesis genes and members of the *SAUR* (SMALL AUXIN UP RNA) gene family, leading to cell elongation and phenotypes such as elongated hypocotyls (Sun et al., 2016). PIF4 also interacts with MRG1/2 (MORF-RELATED GENE 1/2) to facilitate transcription of heat-responsive genes through histone modifications; concurrently, *MRG2* enhances its regulatory function by stabilizing the PIF4 protein (Zhou et al., 2024). Furthermore, HSFA1s (HEAT SHOCK TRANSCRIPTION FACTOR A1s) promote thermomorphogenesis during high daytime temperatures by stabilizing PIF4 (Tan et al., 2023). HSFB2a/B2b (HEAT SHOCK TRANSCRIPTION FACTOR B2a/B2b) are negative regulators of heat stress responses. They undergo ubiquitin-mediated degradation by interacting with XBAT31 (XB3 ORTHOLOG 1 IN ARABIDOPSIS THALIANA), an E3 ligase. This enhances the expression of heat stress-responsive genes and improves plant thermotolerance (Zhang et al., 2024). MicroRNAs (miRNAs) also play a critical role in the regulation of plant growth and various stress response processes (Huang et al., 2025). During heat stress, most miRNAs enhance nuclear-localization of HYL1 (HYPONASTIC LEAVES 1) to induce plant tolerance (Cao et al., 2025). The SUMO E3 ligase SIZ1 participates in the post-transcriptional regulation of thermomorphogenesis by mediating SUMOylation of CPSF100 (CLEAVAGE AND POLYADENYLATION SPECIFICITY FACTOR 100), which in turn modulates the poly(A) site usage of key thermomorphogenesis-related genes (Yu et al., 2024). When exposed to higher temperatures, PIF4 accumulates in the stomatal precursors and binds to the promoter of *SPCH* (SPEECHLESS), a master regulator of stomatal lineage initiation, thereby repressing SPCH expression and reducing the formation of stomata (Lau et al., 2018). The transcription factor *HSFA1b* functions as a heat sensor by modulating stomatal movement under high-temperature stress through inhibition of the kinase activity of OST1 (OPEN STOMATA 1) via its intrinsic adenylate cyclase activity (Zhang et al., 2025). These genes and their interactions collectively form a complex regulatory network that precisely modulates thermomorphogenesis in *Arabidopsis thaliana*.

Thermomorphogenesis in *Arabidopsis thaliana* during both day and night is triggered by the accumulation of PIF4, a central temperature regulator induced by warm temperatures. During the day, PIF4 requires specific stabilization to avoid degradation promoted by active PHYB (Foreman et al., 2010). This stabilization of PIF4 during the day depends on its interaction with HMR, a transcription activator (Qiu et al., 2019). A forward genetic screen using suppressors of the *hmr-22* allele resulted in the identification of a novel daytime temperature signaling component called as *RCB*, which is involved in the modulation of chloroplast biogenesis (Qiu et al., 2021).

In *Arabidopsis thaliana*, non-catalytic thioredoxins are by-products of NUCLEAR CONTROL OF PEP (NCP) activity. They perform distinct and crucial roles in phytochrome signaling across seed plants (Yang et al., 2019). RCB, also known as MRL7, is a thioredoxin, which acts in concert with HMR to initiate thermomorphogenesis by selectively stabilizing PIF4 during the daytime (Qiu et al., 2019, 2021). Plants contain six primary types of thioredoxin proteins (Trx-f, Trx-m, Trx-x, Trx-y, Trx-h, and Trx-o). Different plant cell compartments contain distinct types of thioredoxins. For example, chloroplasts in plants contain Trx-f, Trx-m, Trx-x, and Trx-y (Yua et al., 2014).

Chloroplasts play a critical role in plant responses to environmental stresses (Gan et al., 2019). Under stressful conditions, chloroplasts mediate retrograde signaling and transmit information from the plastids to the nucleus (Song et al., 2021), thereby optimizing nuclear gene expression according to the environmental conditions (Leister et al., 2017). Therefore, chloroplasts regulate transcription and translation of several nuclear genes and modulate specific adaptive responses to environmental stresses. These regulatory mechanisms help plants to survive and grow under stressful conditions. Chloroplast development is a complex process involving multiple proteins. MRL7 is a chloroplast-associated protein in *Arabidopsis thaliana.* MRL7 performs essential functions in chloroplast development and in plastid gene expression (Qiao et al., 2011).

*Karelinia caspica* is a perennial herb belonging to the genus *Karelinia* of the Asteraceae family. It thrives at the edge of deserts and is characterized by flat and distinctly succulent leaves and well-developed water-storage tissues. Therefore, it demonstrates high tolerance to elevated temperatures and drought (Zhang et al., 2014). *Karelinia caspica* can withstand temperatures exceeding 40°C. The inflection point in its tolerance duration at 45°C is approximately 8 hours, whereas its maximum tolerance duration at 50°C is about 1 hour. However, the molecular mechanisms underlying its adaptation to extreme environments remain unclear (Wang et al., 2021). *RCB* gene, a key player in the thermomorphogenesis mechanisms in *Arabidopsis thaliana*, is conserved across plant species and plays a critical role in chloroplast development and temperature signal transduction (Qiu et al., 2021). In *Arabidopsis thaliana*, RCB modulates thermomorphogenesis by synergizing with HEMERA to stabilize PIF4. As a desert plant, *Karelinia caspica* persists in an environment characterized by intense light and extreme temperatures year-round. Therefore, it requires robust chloroplast biogenesis regulatory machinery to maintain photosynthetic efficiency (Zeng et al., 2021). Our transcriptome analysis of *Karelinia caspica* under high-temperature stress demonstrates upregulation of *KcRCB*. Therefore, we hypothesized that *KcRCB* regulates thermotolerance in *Karelinia caspica*.

There have been significant advances in plant thermotolerance research across diverse plant taxa (Kan et al., 2023). However, mechanistic insights into heat adaptation in the desert plant *Karelinia caspica* are unclear. Discovery of the *RCB* gene has facilitated further investigations into the regulatory networks underlying thermotolerance in *Karelinia caspica*. As a keystone species at desert margins, *K. caspica* excels in desert edge stabilization and plays a pivotal role in mitigating land desertification through its ecological functions. In this study, we cloned the *KcRCB* gene and analyzed its expression using the high-temperature transcriptome dataset of *Karelinia caspica*. To evaluate the high-temperature tolerance of *KcRCB* from *Karelinia caspica*, we expressed *KcRCB* in *Arabidopsis thaliana* and cotton and performed physiological experiments.

## 2 Results

### 2.1 Cloning and analysis of the *KcRCB* gene based on transcriptome data of high-temperature-treated *Karelinia caspica*

The transcriptome of high temperature-treated *Karelinia caspica* was compared with the *RCB* gene sequence from *Arabidopsis thaliana* to identify the homologous gene. The full-length *KcRCB* coding sequence (CDS) was amplified by PCR. The *KcRCB* CDS was 1005 bp in length and encoded 334 amino acids (**Supplementary Figure 1**). Based on the amino acid composition, molecular formula of KcRCB protein was predicted to be C_1727_H_2703_N_487_O_526_ S_9_ by ProtParam. Its molecular mass was 38,992.95 and the theoretical isoelectric point (IEP) was 5.57. It consisted of 65 positively charged residues and 5.57 negatively charged residues. ProtScale analysis showed that the KcRCB protein had a maximum hydrophobicity value of 2.144 and a maximum hydrophilicity value of -3.678, indicating that most of its amino acids were hydrophilic.

**Figure 1:**
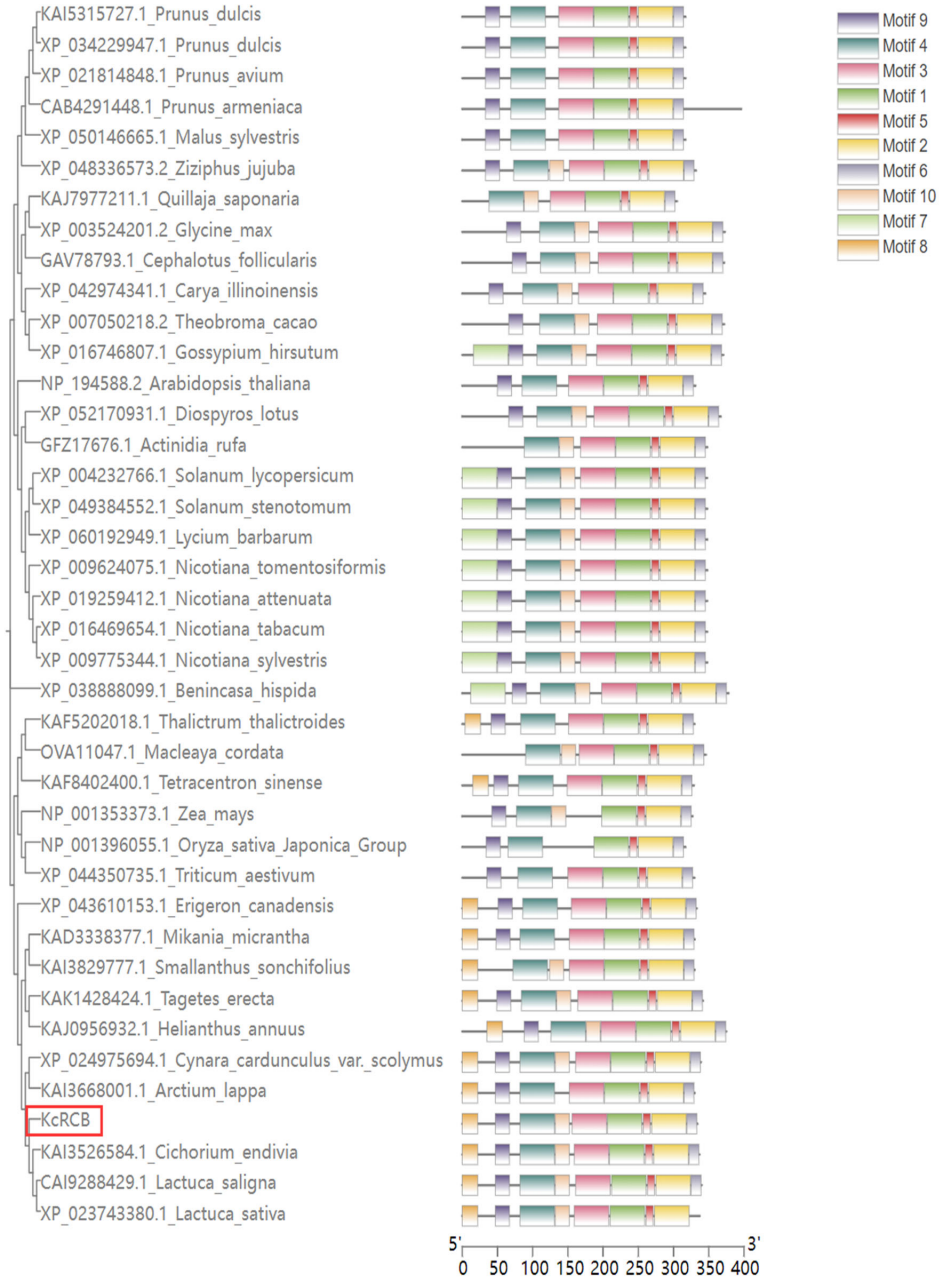
Phylogenetic analysis of RCB proteins in different plant species and identification of protein structure motifs.

Phylogenetic tree construction and conserved domain analysis demonstrated high sequence identify of the amino acid sequence of KcRCB with the RCB proteins of various plant species, including *Lactuca sativa* (82.49% identity), *Cichorium endivia* (81.90% identity), *Cynara cardunculus* (79.06% identity), *Mikania micrantha* (78.81% identity), *Arctium lappa* (78.44% identity), *Tagetes erecta* (77.19%), *Nicotiana tomentosiformis* (70.53%), and *Arabidopsis thaliana* (67.05% identity). Phylogenetic analysis using MEGA 11.0 for sequence alignment combined with MEME-based motif prediction demonstrated that KcRCB was most closely related with the RCB proteins from *Lactuca sativa* and *Cichorium endivia* (Asteraceae). KcRCB contains 9 conserved motifs. Among these, motifs 8 and 10 are additional to those found in the RCB protein from *Arabidopsis thaliana*. Compared with the RCB protein from cotton, KcRCB possesses an extra motif 8 but lacks motif 7; compared to the soybean RCB, it contains additional motifs 7 and 8; compared to maize RCB, it has extra motifs 3, 7, and 8. These differences in motif composition suggest potential functional divergence of RCB proteins across different species (**Figure 1**).

**Figure 2:**
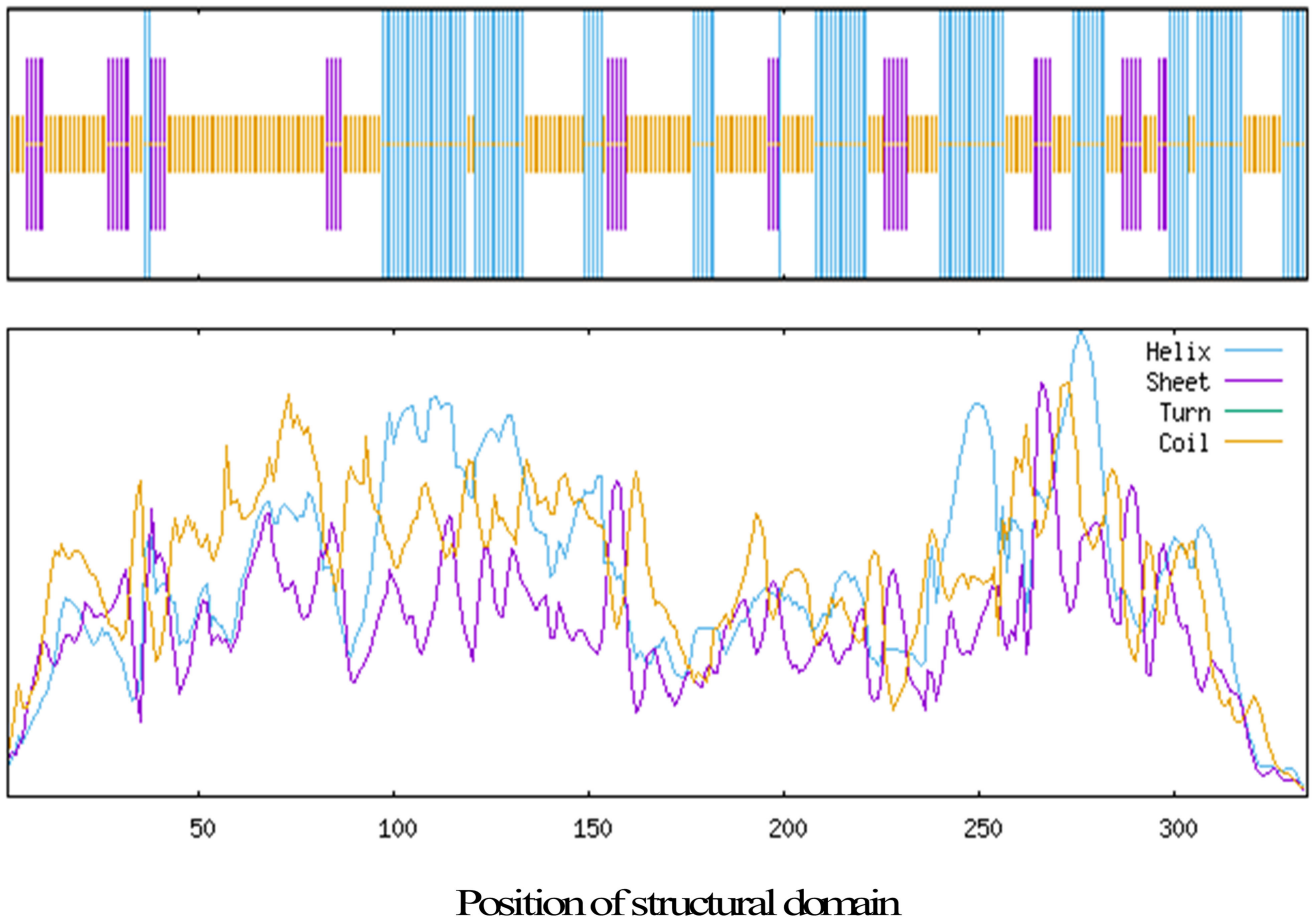
Prediction of the secondary structure of KcRCB.

We then analyzed the secondary structure of the KcRCB protein using SignalP 5.0 and TMHMM online tools and found that both signal peptides and transmembrane domains were absent. SOPMA-based secondary structure prediction showed that KcRCB protein primarily consists of 49.10% random coils, followed by α-helices at 32.04%, extended strands at 14.37%, and β-turns at 4.49% (**Figure 2**).

Domain architecture analysis of KcRCB using InterPro and Prosite identified a thioredoxin-like domain, which shared homology with RCB orthologs from other plant species (**Supplementary Figure 2**). Sequence alignment of KcRCB with RCB proteins from ten additional plant species (**Supplementary File**) confirmed high conservation of this thioredoxin-like domain, despite several species-specific amino acid variations as follows within the domain: (1) KcRCB at position 236 contains aspartic acid (an acidic residue), whereas all other analyzed species possess a basic amino acid at this site; (2) amino acid at position 257 in KcRCB and tobacco homologs is occupied by serine (a hydroxyl-containing residue) compared to asparagine (an amide) in *Arabidopsis thaliana*; (3) glutamine (an amide) at position 302 in KcRCB is substituted by glutamate, serine, threonine, proline, leucine, or lysine in other homologs; (4) lysine (a basic residue) at position 306 in KcRCB is replaced by threonine (hydroxyl-containing) in *Arabidopsis thaliana* and tobacco RCBs, and by serine (hydroxyl-containing) or methionine (sulfur-containing) in other species. These conserved domain variations may contribute to functional divergence of RCB proteins across plant species.

### 2.2 Analysis of the expression pattern of *KcRCB* in *Karelinia caspia* (Pall.) Less.

**Figure 3:**
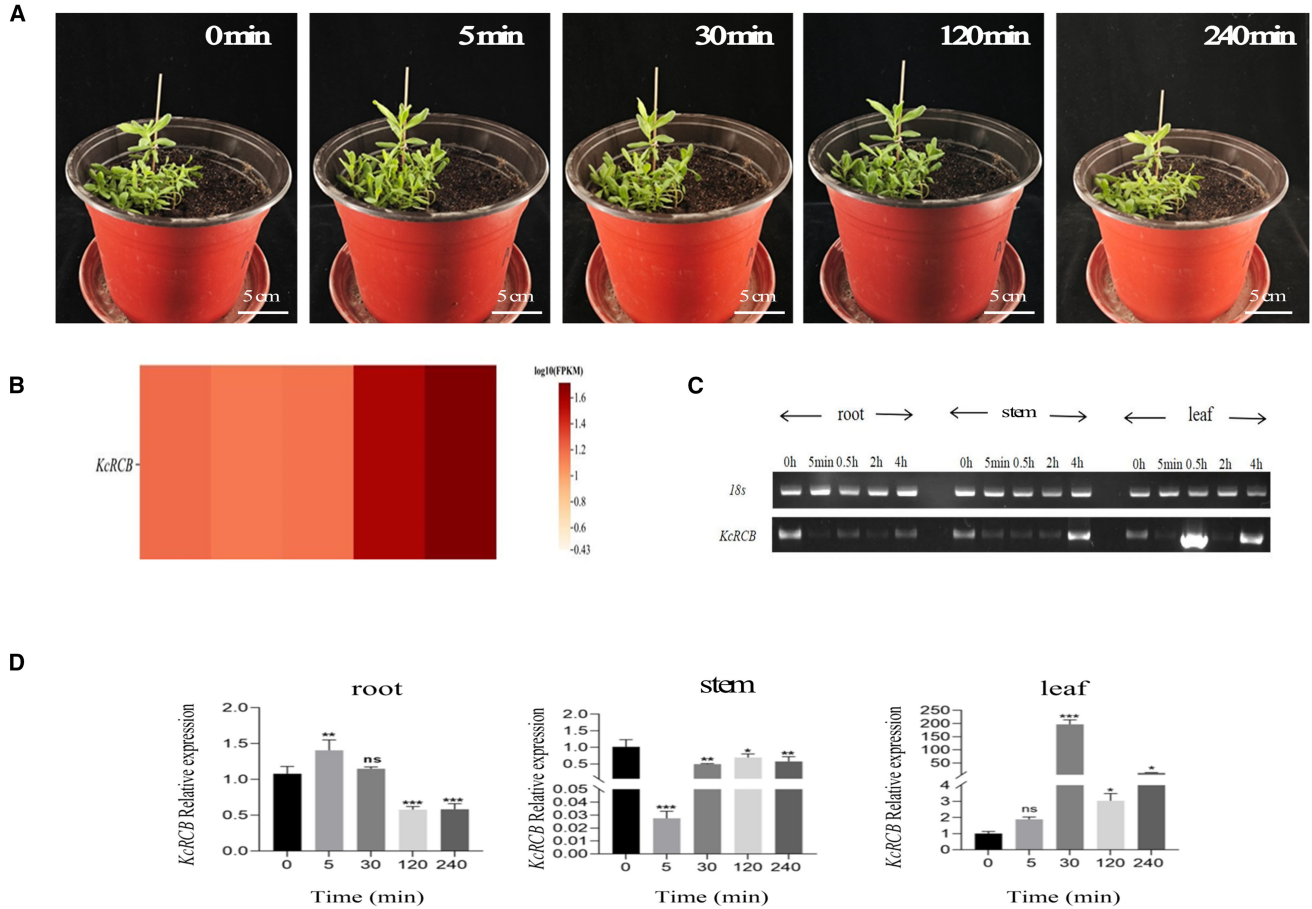
Phenotypic analysis of *Karelinia caspia* during 45°C heat treatment and the expression profile of *KcRCB.* **(A)** Representative images of *Karelinia caspica* phenotype at 0, 5, 30, 120, and 240 min of heat treatment at 45°C. **(B)** Heatmap of *KcRCB* expression in *Karelinia caspica* at 0, 5, 30, 120, and 240 min of heat treatment at 45°C. **(C)** RT-PCR analysis of *KcRCB* gene expression in the root, stem, and leaf tissues of *Karelinia caspica* at different time points during heat treatment at 45°C. **(D)** qRT-PCR analysis of *KcRCB* gene expression in the root, stem, and leaf tissues of *Karelinia caspica* at different time points during heat treatment at 45°C. Data are presented as the mean ± standard deviation (SD) of three biological replicates. ns, no significant difference (*P > 0.05*), *P < 0.05, **P < 0.01, ***P < 0.001.

Phenotypic changes in the *Karelinia caspica* seedlings under a high temperature stress of 45°C are shown in **Figure 3A**. After 5 min of high temperature treatment, *Karelinia caspic*a leaves began to exhibit thermomorphic changes and demonstrated upturning. After 30 min of high temperature treatment, *Karelinia caspica* exhibited more obvious thermomorphic changes with most of the leaves gathering at the center. At 120 min of high temperature treatment, the leaves began to spread slowly. By 240 min of high temperature treatment, the leaves spread to a degree close to the untreated state.

Subsequently, we performed transcriptome sequencing of the high temperature-treated *Karelinia caspica* leaves and used the heatmap to evaluate *KcRCB* gene expression. The calculated FPKM value of *KcRCB* showed dynamic changes over time in the leaves of plants subjected to heat stress 45°C. As shown in **Figure 3B**, FPKM of *KcRCB* initially decreased from 1.21 to 1.16 at 5 min post-treatment, followed by a gradual increase to 1.18 at 30 min, 1.58 at 120 min, and 1.76 at 240 min.

We then performed RT-PCR expression analysis of *Karelinia caspica* plants subjected to high-temperature stress. *KcRCB* exhibited enhanced heat tolerance at 45°C, especially in the leaf tissues. Overall, *KcRCB* expression was lower in the roots compared to the control, thereby indicating that *KcRCB* did not function significantly in the roots. In the stem, *KcRCB* expression decreased initially, followed by a gradual increase with the time of higher temperature treatment. In the leaves, *KcRCB* gene expression showed a fluctuating trend with increasing treatment time and reached maximum expression at 30 min (**Figure 3C**).

**Figure 4:**
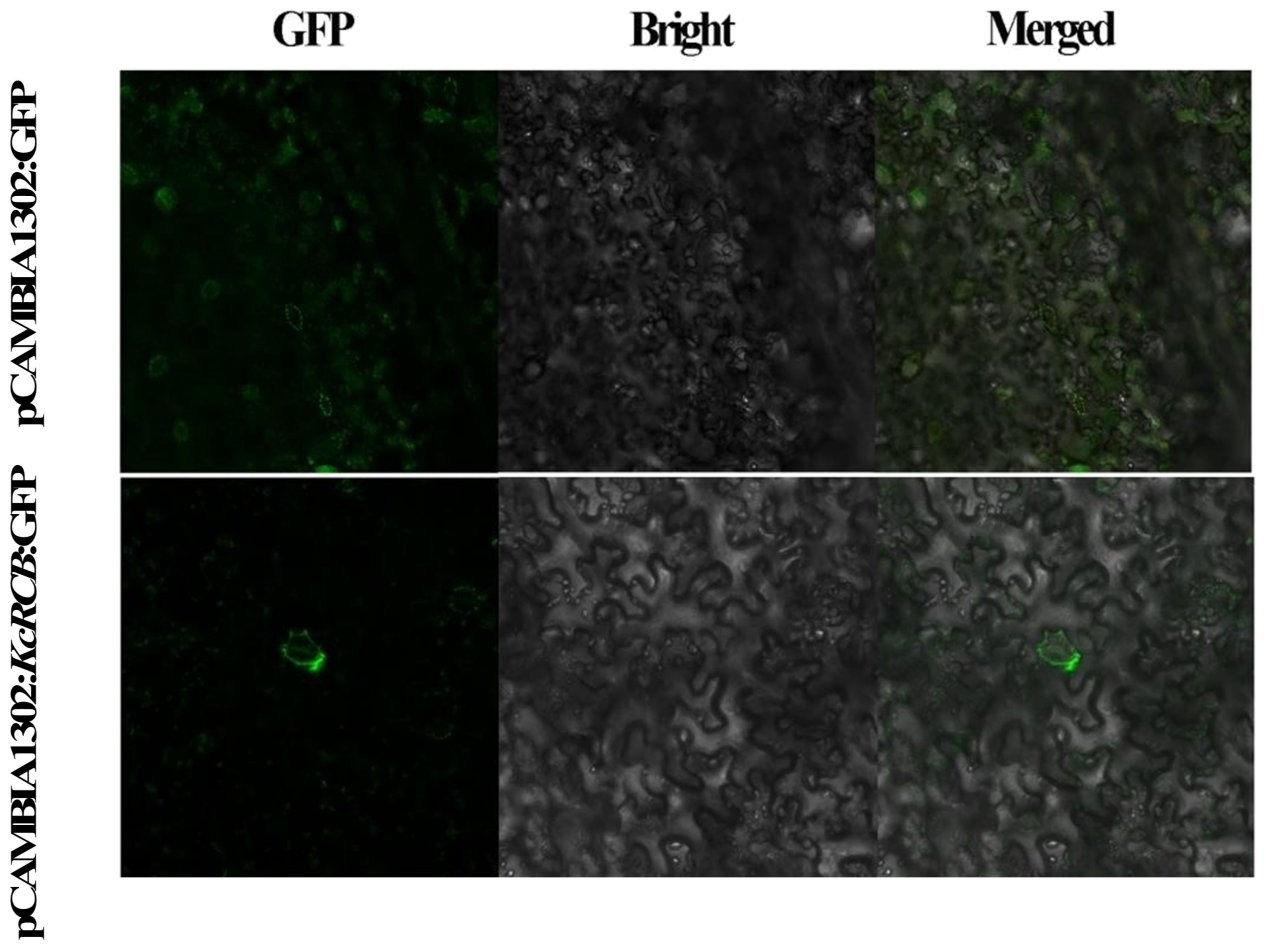
Subcellular localization of KcRCB in *Karelinia caspia*.

We then performed real-time quantitative PCR (RT-qPCR) analysis and found that the relative expression of *KcRCB* was low in the roots and decreased with increasing high temperature treatment time. In the stem, relative expression of *KcRCB* decreased initially, but then increased as the time of higher temperature treatment progressed. The relative expression levels of *KcRCB* transcripts in the stem were significantly lower than those before treatment. In the leaves, relative expression levels of *KcRCB* transcripts increased at 30 min of treatment, then decreased briefly at 120 min, and increased again at 240 min to reach a maximum. Relative expression of *KcRCB* in the leaves reached maximum level at 30 min of treatment, then decreased at 120 min, and subsequently increased again to nearly the same maximal level at 240 min (**Figure 3D**). This demonstrated that *KcRCB* responded significantly to high temperatures in the leaves. The subcellular localization analysis in tobacco demonstrated that *KcRCB* was localized in the leaf stomatal guard cell membrane (**Figure 4**).

### 2.3 Overexpression of *KcRCB* enhances the heat tolerance of *Arabidopsis thaliana*

**Figure 5:**
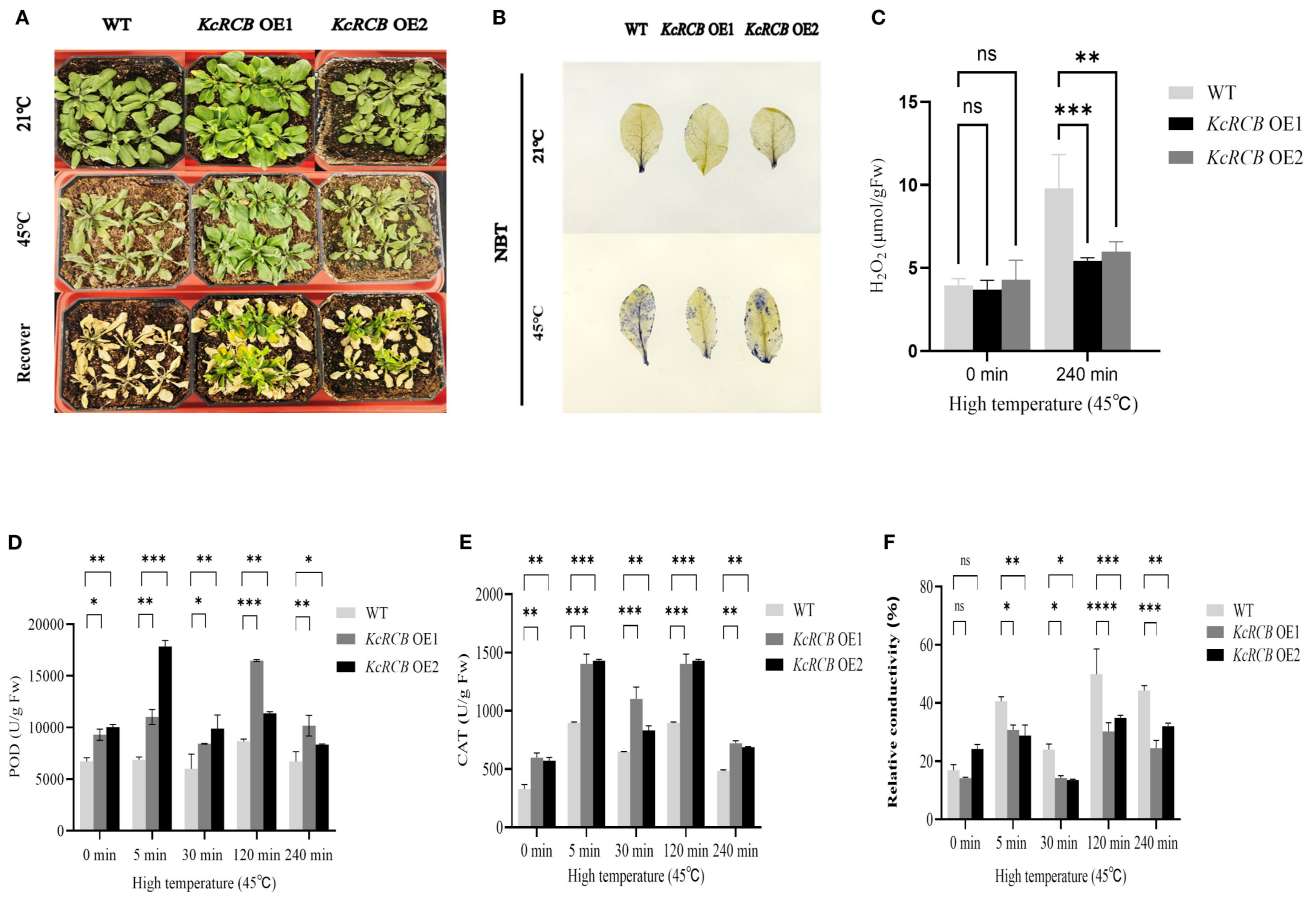
*KcRCB* overexpression enhances heat tolerance in *Arabidopsis thaliana.* ***(*A*)*** Comparison of phenotypes in the wild-type *Arabidopsis thaliana* and *KcRCB*-overexpressing lines (OE1, OE2) under normal cultivation, heat treatment at 45°C for 6 h, and 3 days of recovery after heat treatment. **(B)** NBT staining of wild-type *Arabidopsis thaliana* and *KcRCB* overexpression lines (OE1, OE2) leaves under normal culture conditions and after heat treatment at 45°C for 240 min. **(C)** Estimation of H_2_O_2_ content in the wild-type and *KcRCB*-transgenic *Arabidopsis thaliana* under normal conditions and heat treatment at 45°C for 240 min. **(D-E)** Estimation of **(D)** peroxidase (POD) and **(E)** Catalase (CAT) activity activities in the wild-type and *KcRCB*-transgenic *Arabidopsis thaliana* under normal conditions and heat treatment at 45°C. **(F)** Relative conductivity measurements in the wild-type and *KcRCB*-transgenic *Arabidopsis thaliana* under normal conditions and heat treatment at 45°C. Data are presented as the mean ± standard deviation (SD) of three biological replicates. “ns”, no significant difference (*P > 0.05*), *P < 0.05, **P < 0.01, ***P < 0.001, ****P < 0.0001.

Wild-type and *KcRCB*-overexpressing (*KcRCB*-OE) transgenic *Arabidopsis thaliana* plants were cultivated until they reached the seedling stage. Then, they were placed in a 45°C artificial climate chamber to undergo high-temperature stress treatment. As shown in **Figure 5A**, there were no differences in the growth patterns between the wild-type and *KcRCB* OE2 *Arabidopsis thaliana* plants cultured at 21°C. However, *KcRCB* OE1 *Arabidopsis thaliana* plants grew relatively luxuriantly. After 6 h of high-temperature stress, the leaves of wild-type plants showed severe wilting, but the two *KcRCB*-overexpression strains (OE1 and OE2) showed less wilting than the wild-type. After high-temperature treatment for 6 h, the wild-type and *KcRCB*-OE *Arabidopsis thaliana* plants were transferred to a 21°C incubator and cultivated normally for three days. We observed that all the *KcRCB*-overexpressing plants grew new leaves, but the wild-type *Arabidopsis thaliana* plants did not show recovery.

The *KcRCB*-OE transgenic plants (OE1 and OE2) and wild-type *Arabidopsis thaliana* plants were transplanted into soil and cultivated until the seedling stage. They were subjected to heat stress treatment at 45°C. Then, we performed NBT staining and H_2_O_2_ estimation in the leaves of plants cultivated at 21°C (control) and those subjected to 240 min heat treatment at 45°C. Furthermore, we collected leaf tissues from each group at 0, 5, 30, 120, and 240 min after the onset of heat treatment and measured catalase (CAT) and peroxidase (POD) activities, as well as relative electrical conductivity.

NBT staining did not show significant differences between wild-type and *KcRCB*-OE plants under normal cultivation conditions (**Figure 5B**). However, after 240 min of heat stress treatment, we observed significant concentrations of superoxide anions in the entire leaf of the wild-type plants, but significantly lower levels of superoxide anions in the leaves of *KcRCB* OE1 and *KcRCB* OE2 *Arabidopsis thaliana* plants. Moreover, ROS levels were slightly higher in the leaves of *KcRCB* OE2 plants than those of the *KcRCB* OE1 plants.

We then estimated H_2_O_2_ levels in the wild-type and *KcRCB*-OE *Arabidopsis thaliana* leaves before and after high-temperature treatment (**Figure 5C**). We did not observe any significant differences in the H_2_O_2_ levels content between the leaves of the wild-type and *KcRCB*-OE lines before high-temperature treatment. However, after 240 min of high-temperature stress, the H_2_O_2_ content in the leaves of the wild-type *Arabidopsis thaliana* plants was significantly higher than that of the overexpression lines. Moreover, H_2_O_2_ content in the leaves of the *KcRCB* OE2 plants was slightly higher than those of the *KcRCB* OE1 plants.

POD activity reflects plant tolerance to various stresses. POD activity significantly increased at 5 and 120 min and decreased at 240 min in both the wild-type and *KcRCB*-OE lines (**Figure 5D**). Catalase activity of the transgenic plants overexpressing *KcRCB* was consistently higher than those of the wild type, thereby indicating enhanced antioxidant capacity (**Figure 5E**). Before undergoing high-temperature treatment, there were no significant differences in the relative conductivity between the overexpression lines and the wild type. However, relative conductivity in both the wild-type and *KcRCB*-OE lines increased after 5 min of heat treatment, then showed a decline at 30 min, and subsequently increased again at 120 min and 240 min of heat treatment (**Figure 5F**). The relative electrical conductivity of the two overexpression plants was significantly lower than that of the wild type. This suggested that the electrolyte leakage in the overexpression plants was lower than in the wild type plants.

### 2.4 Overexpression of *KcRCB* reduces cotton leaf damage

**Figure 6:**
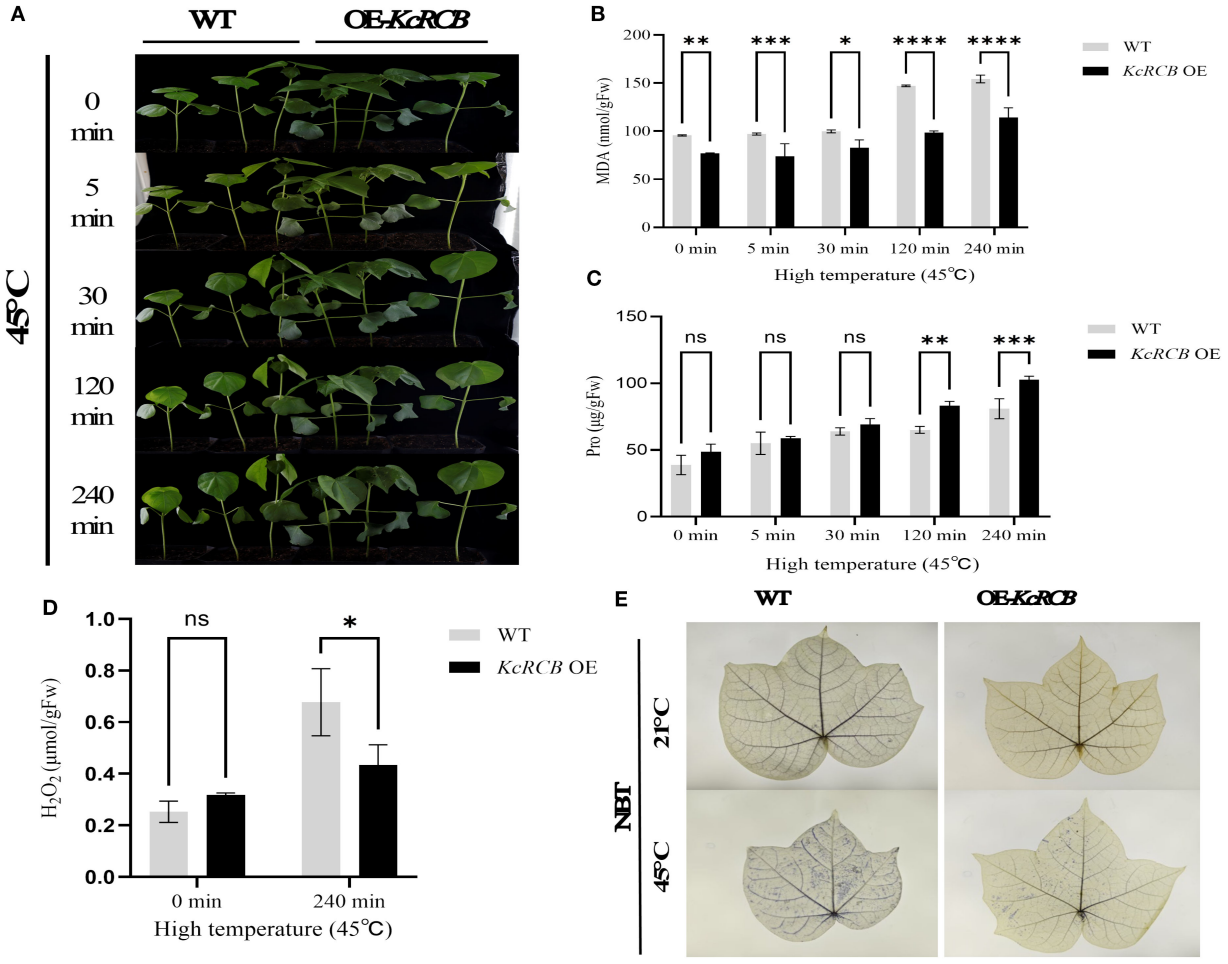
*KcRCB* overexpression alleviates heat-induced damage in the cotton leaves. **(A)** Phenotypic changes in the *KcRCB*-overexpressing and non-transgenic cotton plants subjected to heat treatment at 45°C for 0, 5, 30, 120, and 240 min. **(B)** Estimation of malondialdehyde (MDA) content in the leaves of *KcRCB*-overexpressing and non-transgenic cotton plants subjected to heat treatment at 45°C. **(C)** Estimation of hydrogen peroxide (H_2_O_2_) content in the leaves of *KcRCB*-overexpressing and non-transgenic cotton plants subjected to heat treatment at 45°C. **(D)** Nitroblue tetrazolium (NBT) staining of leaves from the *KcRCB*-overexpressing and non-transgenic cotton plants subjected to heat treatment at 45°C. Data are presented as the mean ± standard deviation (SD) of three biological replicates. “ns”, no significant difference (*P > 0.05*), *P< 0.05, **P< 0.01, ***P< 0.001, ****P< 0.0001. Fw: fresh weight.

In this study, the cotton cultivar “Tahe 2” employed was developed by Xinjiang Tarim River Seed Industry Co., Ltd. This cultivar is an medium-maturity, non-transgenic conventional upland cotton with medium fiber, exhibiting resistance to *Fusarium* wilt and tolerance to *Verticillium* wilt. We obtained transgenic lines overexpressing the *KcRCB* gene by tissue culturing and verified their high-temperature tolerance. The *KcRCB*-overexpressing plants and control plants were subjected to heat stress treatment at 45°C in an artificial climate chamber. As shown in **Figure 6A**, we did not observe phenotypic differences between the *KcRCB*-overexpressing and control plants at room temperature. After 30 min of cultivation in a 45°C incubator, the control plants began exhibited leaf drooping and mild leaf wilting, whereas *KcRCB*-overexpressing plants showed slightly drooping leaves. After 120 min of heat stress, the leaf drooping angle of the control group increased significantly with curling, whereas the leaf drooping angle of the *KcRCB*-overexpressing plants increased slightly. After 240 min of heat stress, the leaf drooping angle of the control plants approached 90°, whereas leaf drooping angle of the *KcRCB*-overexpressing plants showed a slight increase in drooping angle.

Subsequently, we measured MDA activity and proline (Pro) content in the control and *KcRCB*-overexpressing plants under heat stress at different treatment times. MDA activity in both the control and *KcRCB*-overexpressing plants exhibited an upward trend with increasing treatment time. However, the control group showed a significant increase in MDA activity after 120 min of treatment, whereas *KcRCB*-overexpressing plants showed only a mild increase in MDA activity. Throughout the entire heat stress period, MDA activity in the *KcRCB*-overexpressing plants was lower than in the control plants and reached significant levels at 120 min and 240 min (**Figure 6B**). MDA activity results suggested that plasma membrane damage during heat stress was significantly lower in the *KcRCB*-overexpressing plants.

During the entire treatment period, proline (Pro) content showed an upward trend in both the control and *KcRCB*-overexpressing plants. In the early stage of heat treatment, Pro content was similar in both between the control and *KcRCB*-overexpressing plants, but increased significantly after 120 min of treatment. Throughout the heat stress period, Pro content in the *KcRCB*-overexpressing plants was consistently higher than in the control group and showed significant differences from the control group after 120 min (**Figure 6C**). The Pro content data demonstrated that stress tolerance was significantly higher in the *KcRCB*-overexpressing plants than in the control group.

After 240 min of heat stress, we performed NBT staining and estimated H_2_O_2_ content in the leaves of the *KcRCB*-overexpressing and control plants. NBT staining results showed that both the control and the *KcRCB*-overexpressing plants did not show any differences in ROS accumulation or phenotype before heat treatment. However, after heat treatment, the leaves of control plants exhibited darker staining than the *KcRCB*-overexpressing plants, thereby indicating higher ROS accumulation in the control plants (**Figure 6E**).

Estimation of H_2_O_2_ content in the cotton leaves before and after heat treatment (**Figure 6D**) showed that there were no significant differences between the control and *KcRCB*-overexpressing plants prior to heat treatment. However, after 240 min of heat stress, leaves of the control plants exhibited significantly higher H_2_O_2_ levels than those from the *KcRCB*-overexpressing plants. This further validated the NBT staining results and showed significant accumulation of ROS in the control plant leaves after heat stress compared with the *KcRCB*-overexpressing plants.

**Figure 7:**
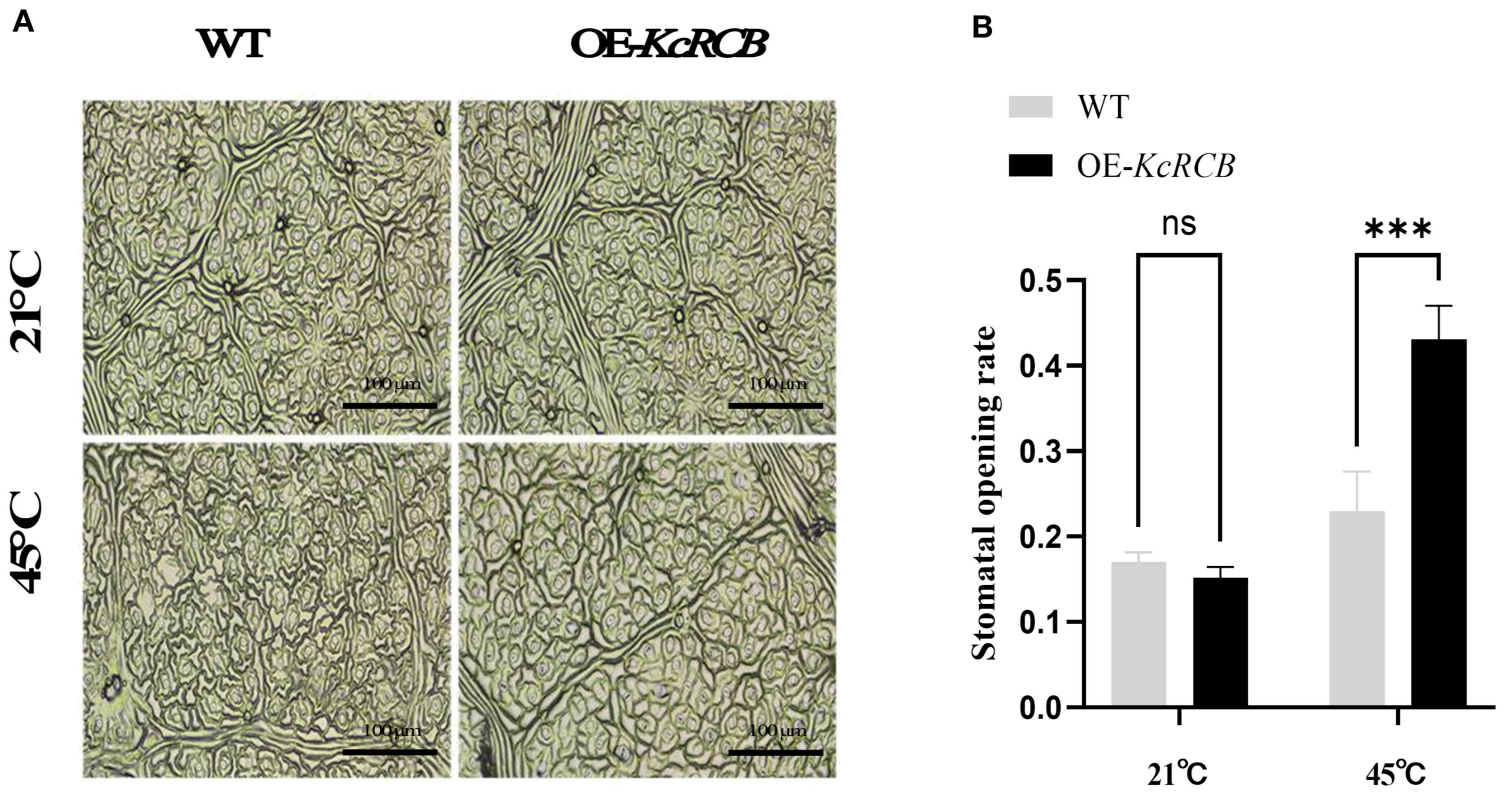
*KcRCB* overexpression promotes stomatal opening in cotton under heat stress. **(A)** Stomatal changes in the leaves of *KcRCB*-overexpressing and non-transgenic cotton plants subjected to heat treatment at 45°C for 240 min. **(B)** Stomatal opening/closing rates in the leaves of *KcRCB*-overexpressing and non-transgenic cotton plants before and after heat treatment at 45°C. Data are presented as the mean ± standard deviation (SD) of three biological replicates. “ns”, no significant difference (*P > 0.05*), ***P < 0.001.

We analyzed the stomatal aperture of the cotton leaves in the *KcRCB*-overexpressing and control plants before and after heat treatment. There were no significant differences in the stomatal aperture of the cotton leaves of the control and *KcRCB*-overexpressing plants cultivated at room temperature. However, after heat stress, the *KcRCB*-overexpressing plants exhibited a higher degree of stomatal opening (**Figure 7A**). Stomatal aperture was quantified by calculating the stomatal opening rate in three biological replicates per group. As shown in **Figure 7B**, there were no significant differences in the stomatal opening rate between the *KcRCB*-overexpressing and the control plants before heat treatment. However, stomatal opening rate in the *KcRCB*-overexpressing plants after 240 min of heat stress was approximately 2-fold higher than that of the control group. These results suggest that overexpression of *KcRCB* enhances stomatal opening under heat stress, thereby promoting transpiration to reduce leaf temperature and mitigate leaf damage.

## 3 Discussion

Our study shows that the KcRCB protein enhances plant tolerance to high temperatures by regulating stomatal opening. Validation experiments in *Arabidopsis thaliana* (model plant) and cotton (cash crop) plants demonstrate that overexpression of the *Karelinia caspica RCB* gene mitigates heat-induced leaf damage, delays the onset of heat stress morphology, and improves plant thermotolerance.

*Karelinia caspica* is a perennial herbaceous plant belonging to the genus *Karelinia* in the Asteraceae family. It primarily inhabits sand dunes, Gobi deserts, saline-alkaline lands, and the vicinity of reed marshes and paddy fields, often forming large-scale populations. This species exhibits strong stress resistance traits, including high tolerance to salinity, drought, and extreme temperatures. In China, *Karelinia caspica* is mainly distributed in the desert regions. Its flat and fleshy leaves are well-adapted for water storage and retention to thrive in arid conditions. *Karelinia caspica* can flower normally in the Taklamakan Desert when the temperatures exceeds 45°C in the summer, thereby signifying its exceptional heat tolerance (Wang et al., 2021). Our research findings show that the *KcRCB* gene expression levels in heat-stressed *Karelinia caspica* show a decreasing trend first but increase significantly with prolonged exposure to high temperatures. The high expression of *KcRCB* is synchronized with the heat-tolerant phenotype. This suggests that *KcRCB* is involved in the response of *Karelinia caspica* to high-temperature stress.

### 3.1 Identification and analysis of *KcRCB*

Current research on *RCB* genes is limited to the model plant *Arabidopsis thaliana* (Yoo et al., 2019; Qiu et al., 2021). In this study, we cloned the *KcRCB* gene using transcriptomic data from *Karelinia caspica*, based on the sequence of *Arabidopsis thaliana RCB* (AT4G28590). Domain analysis, phylogenetic tree construction, and motif prediction analysis showed that the sequence of the RCB protein was conserved and belonged to the MRL7 thioredoxin-like superfamily, consistent with findings in *Arabidopsis thaliana* (Yang et al., 2019). Thioredoxins are ubiquitous and thermostable proteins that function as hydrogen carriers. *KcRCB* exhibits amino acid variations compared to other plant species in the conserved region. These variations may contribute to differences in thermostability. These motif variations across species may also be responsible for the functional divergence of *KcRCB* compared to other plant RCB proteins.

In *Arabidopsis thaliana*, MRL7 was initially identified as a regulator of chloroplast growth and development, influencing plastid gene expression (Qiao et al., 2011). Subsequent studies demonstrated that *Arabidopsis thaliana* Early Chloroplast Biogenesis 1 (*AtECB1*), an allelic variant of MRL7 encoded a thioredoxin-like fold protein, and modulated the activity of the plastid-encoded RNA polymerase (PEP) and chloroplast biogenesis (Yua et al., 2014). Furthermore, immunological analyses showed that Suppressor of Variegation 4 (SVR4, also known as MRL7) was a component of the Transcriptionally Active Chromosome (TAC) complex in the barley chloroplasts. Gene expression studies further demonstrated that SVR4 and SVR4-like proteins are essential for the normal functioning of the plastid transcriptional machinery (Powikrowska et al., 2013).

Recent studies have shown that the RCB protein is a dual-targeted nucleus/plastid component required for *PEP* assembly. RCB interacts with phytochromes in the nucleus and promotes degradation of transcription factors PIF1 and PIF3, which localize to the photobodies and regulate *PhAPG* (plastid-encoded photosynthetic genes) expression levels (Yoo et al., 2019). RCB-dependent PIF degradation in the nucleus signals PEP assembly and *PhAPG* expression in the plastids. Emerging evidence suggests that RCB acts as a novel temperature signaling component and interacts with HMR to selectively stabilize PIF4 during the day, thereby initiating thermomorphogenesis.

We then performed RT-PCR and qRT-PCR analyses to investigate the expression patterns of *KcRCB* in various tissues of *Karelinia caspica* under heat stress. *KcRCB* is predominantly expressed in the leaves and shows time-dependent increase in the *KcRCB* transcript levels during heat treatment. Furthermore, subcellular localization data demonstrated *KcRCB* accumulation in the guard cells surrounding the stomatal pores. Based on these data, we hypothesize that *KcRCB* contributes to temperature regulation by enhancing stomatal conductance and transpiration. This mechanism facilitates leaf cooling and alleviates heat-induced damage (Zhou et al., 2023). Collectively, these findings indicate that *KcRCB* plays a positive regulatory role in mediating thermotolerance in *Karelinia caspica.*

### 3.2 *KcRCB* enhances heat tolerance in *Arabidopsis thaliana*

To further investigate the function of *KcRCB*, we conducted preliminary functional validation in the model plant *Arabidopsis thaliana*. We did not observe significant phenotypic differences between *KcRCB*-overexpressing transgenic plants and non-transgenic controls under normal conditions. However, the *KcRCB*-overexpressing transgenic plants exhibited significantly enhanced thermotolerance during heat stress.

Heat stress commonly affects multiple physiological and biochemical processes in plants, including membrane thermostability, antioxidant enzyme activity, osmotic regulators, and photosynthetic characteristics. During heat stress, excessive accumulation of reactive oxygen species (ROS) such as H_2_O_2_, O_2_^-^, and ·OH, causes significant lipid peroxidation and electrolyte leakage, thereby damaging the plants significantly (Dvořák et al., 2021). In our study, transgenic *Arabidopsis thaliana* showed lower malondialdehyde (MDA) concentration and relatively conductivity than the wild-type plants under heat stress, thereby indicating improved protection of the leaf cell membranes. Furthermore, activities of antioxidant enzymes catalase (CAT) and peroxidase (POD) were significantly higher in the *KcRCB*-overexpressing transgenic plants than in wild-type controls. NBT staining and H_2_O_2_ content assays also demonstrated excessive ROS accumulation in the wild-type plants but lower ROS levels in the *KcRCB*-overexpressing transgenic lines after heat stress. These results suggest that *KcRCB* mitigates heat-induced damage by enhancing ROS scavenging.

### 3.3 Overexpression of *KcRCB* reduces cotton leaf damage

Our study showed that *KcRCB* overexpression enhanced heat tolerance by decreasing membrane damage and increasing ROS scavenging in *Arabidopsis thaliana*. To verify whether KcRCB is stably expressed in other plant species, we further verified the effects of KcRCB expression in cotton. Transgenic cotton was obtained by Agrobacterium-mediated transformation via tissue culturing. We selected *KcRCB* overexpressing cotton as the experimental group for analyzing high-temperature stress at 45°C. Phenotypic observations demonstrated that the wilting and recurved angle of cotton leaves in the high-temperature treated control group were significantly higher than in the corresponding experimental group. Furthermore, NBT staining and hydrogen peroxide estimations showed that the ROS levels were significantly elevated in the control group than in the *KcRCB* overexpressed plants. MDA analysis demonstrated that *KcRCB* overexpression reduced membrane damage in the leaf cells.

### 3.4 KcRCB is localized to the leaf stomatal guard cell membrane

Heat-tolerant plants mitigate the effects of high-temperature stress by increasing transpiration rates to facilitate efficient leaf cooling. This adaptive strategy is attributed to increased stomatal density and total stomatal pore area per leaf, which enables optimal gas exchange and thermoregulation. By modulating stomatal density, aperture size, and morphology, heat-tolerant plants protect chloroplast structure and function while maintaining membrane thermostability during heat stress (Hao et al., 2019).

In our study, statistical analysis of stomatal aperture and density in the transgenic cotton plants overexpressing *KcRCB* demonstrated a significant increase in the stomatal opening during heat stress compared to the wild-type control plants, which exhibited widespread stomatal closure. These findings suggest that *KcRCB* enhances thermotolerance by promoting stomatal conductance, thereby increasing transpiration and reducing leaf temperature. This mechanism effectively safeguards the leaves from heat-induced damage and underscores the critical role of *KcRCB* in regulating stomatal dynamics and improving heat resilience in the transgenic cotton plants.

Current knowledge of the molecular mechanisms underlying thermomorphogenesis primarily focuses on the following three aspects: protein-level regulation; expression of *PIF4*; and chromatin state modifications via promoter binding (Quint et al., 2016). The core pathway of thermomorphogenesis is governed by *PIF4* and other regulatory factors, which coordinate plant growth and development in response to temperature and light cues (Proveniers and van Zanten, 2013). High temperatures inhibit stomatal production and alter stomatal morphology. *KcRCB* is localized in the plasma membrane of the leaf guard cells. Previous studies have shown that *RCB* plays a key role in the *PIF4* regulatory pathway (Qiu et al., 2021). As a central component of high-temperature signaling, PIF4 accumulates in the stomatal precursors upon heat exposure and binds to the promoter of *SPCH*. Temperature-activated PIF4 represses *SPCH* expression to limit stomatal production under heat stress (Lau et al., 2018). Future investigations are necessary to determine whether *KcRCB* participates in this regulatory pathway and its potential interactions with the *PIF4* network.

Our study has a few limitations. Although our experiments showed that stomatal opening of the leaves in the *KcRCB*-overexpressing plants was enhanced under high-temperature stress, the molecular mechanisms underlying these effects are not clear. The genomic data of *Karelinia caspica* is not available. Moreover, transcriptomic data is incomplete and chromosomal localization results are unclear. Therefore, it is difficult to perform in-depth investigations of the regulatory network underlying the functions of *KcRCB*. In the future, mining of the complete *Karelinia caspica* transcriptome database is necessary to identify the upstream and downstream genes of the *KcRCB* pathway. The results of our study provide a molecular basis for investigating the regulatory mechanisms of *KcRCB* in adverse conditions.

## 4 Conclusions

In this study, we cloned the chloroplast development-related gene *KcRCB* from *Karelinia caspica*, a plant adapted to extreme environments. The full-length cDNA of *KcRCB* is 1005 bp and encodes 335 amino acids. The amino acid sequence analysis demonstrates a high degree of evolutionary conservation between *KcRCB* and its homologous genes in Asteraceae plants. Subcellular localization experiments suggest that KcRCB is localized in the leaf stomata, but further co-localization assays are necessary to determine its precise localization. Functional analysis demonstrated that overexpression of *KcRCB* significantly improves the heat tolerance of transgenic *Arabidopsis thaliana* and cotton plants by enhancing the stomatal opening of the leaves, thereby, highlighting its important role in plant thermomorphogenesis.

## 5 Materials and methods

### 5.1 Cloning and analysis of the *KcRCB* gene

*KcRCB* was identified through a two-step selection process from the *Karelinia caspica* transcriptome datasets. Initially, genes exhibiting constitutively high expression (top 5% FPKM values in both control and heat-stressed *Karelinia caspica* leaf samples) were prioritized because highly abundant transcripts are typically indicative of core components in essential biological pathways (e.g., chloroplast maintenance). In the next step, we selected differential expressing genes (p < 0.05 under high-temperature stress) among these high-abundance candidates that also display stress-responsive expression patterns. Therefore, we selected genes with putative dual functions in both basal physiological processes as well as in adaptive stress responses. The homologous *KcRCB* gene was cloned by comparing the third-generation full-length transcriptome data of high-temperature-treated *Karelinia caspica* with the *AtRCB* gene, and cloned using the gene-specific primers listed in **Table 1**. The amino acid sequence of *KcRCB* was compared with the RCB protein sequences of other plants using the NCBI BLAST website (https://blast.ncbi.nlm.nih.gov/Blast.cgi). Hydrophobicity of the KcRCB protein was analyzed using the Protscale online tool on the EXPASY website (https://www.expasy.org/). The conserved structural domains of the KcRCB protein were predicted using the Prosite and Interpro online databases (https://www.ebi.ac.uk/interpro/). The physicochemical properties of the KcRCB protein, including isoelectric point (PI) and amino acid composition were predicted using ProtParam tool on the EXPASY website. The secondary structure of KcRCB protein was analyzed using the SOPMA online tool (https://npsa.lyon.inserm.fr/). The motif analysis of the KcRCB protein was performed using MEME (https://meme-suite.org/meme/) to predict the signal peptide. The transmembrane structural domain of the KcRCB protein was predicted by using TMHMM tool (https://services.healthtech.dtu.dk/services/TMHMM-2.0/). A phylogenetic tree was constructed via sequence alignment using MEGA11.0, and the evolutionary relationships, protein phylogenetic analysis, and conserved protein motifs among several plant species with protein sequences homologous to KcRCB were analyzed.

**Table 1:** Primers used in this study.

Primer name	Primer sequence (5’→3’)	Purpose
KcRCBF	5'-GGGTTTGAACCCTTGCAA-3'	Gene clone
KcRCBR	5'-AATTCGAAGCAGAAAATATTC TTACTATATAAAATAT-3'	Gene clone
KcRCB-q- F	5'-TTGAGACAGACCTTGCGTA-3'	Gene expression analysis
KcRCB-q- R	5'-GCACCAACTCATCTGCTTT-3'	Gene expression analysis

### 5.2 Expression pattern of *KcRCB* in *Karelinia caspia* (Pall.) Less.

Seeds of *Karelinia caspia* were sown in a growth substrate composed of nutrient soil and vermiculite at a volume ratio of 3:1. The seeds were then covered with a membrane. The seeds were gently prodded to encourage seedling development. The membrane was removed after two days and the seedlings were cultivated for two months at 28°C, with 16 h of light and 8 h of darkness. *Karelinia caspica* seedlings exhibiting similar growth patterns were then exposed to a temperature of 45°C for 5, 30, 120, and 240 min. Each treatment was repeated thrice. The entire process was photographed. After the treatment, we collected the roots, stems, and leaves for RNA extraction using the RNAprep Pure Plant Kit (Beijing Tiangen Biochemical Technology). Then, reverse transcription was performed to synthesize cDNA using a Easy Script^®^ One-Step gDNA Removal and cDNA Synthesis Kit (Beijing Quan’s Jin Biotechnology). We then estimated the concentration of cDNA samples. RT-PCR analysis was performed to determine the relative expression levels of *KcRCB* in plants treated at 45°C. The PCR products were analyzed by electropherogram and the expression pattern was evaluated based on the brightness of specific bands. The relative expression of *KcRCB* during 45°C treatment was analyzed by real-time fluorescence quantitative PCR (RTqPCR) using Beijing Quan’s Gold Biotechnology PerfectStart^®^ Fast Green qPCR SuperMix. The q-PCR reaction was performed using the KcRCB-q- F and KcRCB-q- R primers (**Table 1**).Data analysis and graphical representation was performed using the GraphPad Prism software.

### 5.3 Subcellular localization of KcRCB in *Nicotiana tabacum* L.

To determine subcellular localization of KcRCB, the target gene was cloned into the pCAMBIA-1302 vector using the 2×Seamless Cloning Mix kit (Beijing Bomade Biologicals) and *KcRCB*-1302 F and *KcRCB*-1302 R primers shown in **Table 1**. The T vector plasmid with KcRCB was used as the template for PCR amplification of the target gene. The pCAMBIA1302 vector was double digested with the Bgl II and Spe I enzymes. The digested product was isolated from the agarose gel and mixed with the PCR amplification product of *KcRCB* according to the manufacturer’s instructions. The ligated product was then incubated at 50°C for 30 min. The ligated product was subsequently transferred into the E. coli DH5α cells and the positive clones were selected. The recombinant plasmid was then electroporated into the GV3101 electrocompetent cells at appropriate conditions. The capacitance of the electroporation cup was set at 25 μF. The resistance of the power supply was 200 ohms, and the voltage was 2400 V. The electroporation cup was quickly inserted into the electro transferring tank and ‘single colony activation’ option was used to identify the positive clones.

To determine the subcellular localization of KcRCB, the transformed Agrobacterium pCAMBIA1302-*KcRCB* was cultured until an OD_600_ of 1.2. Then, the bacterial solution was resuspended with 5 mL of MMA (10 mM MgCl_2_, 150 μM AS, 10 mM MES, adjusted to pH 5.6), and incubated at room temperature for 2–6 h until an OD_600_ of 0.6–0.8 was obtained. Subsequently, we selected 4-week-old Benjamin’s tobacco and injected the bacterial solution with the target genes and the nuclear marker solution with a 1 mL syringe at a ratio of 1:1 into the three upper full-spreading leaves, which were then marked for identification. The darkening of leaves were observed after 2 days, followed by a 2-day period of normal incubation. The leaves were then labeled, harvested, inverted on a slide, and observed under a laser confocal microscope.

### 5.4 Analysis of the high-temperature tolerance in *KcRCB-overexpressing Arabidopsis thaliana* (L.) Heynh.

The *KcRCB* gene was ligated into pONDR221 using the Gateway™ BP Clonase™ Enzyme Mix (Thermo Fisher Scientific). Then, the target gene was cloned into the pK2GW7 overexpression vector and transferred into the GV3101 Electroporation-Competent cells (Shanghai Vidi Biotech) using the Gateway™ LR Clonase™ enzyme mixture.

*Arabidopsis thaliana* was infected using the flower-dipping method (Zhang et al., 2006). At the time of full bloom, the flowers and pods were pollinated and then removed. The flower buds were then infected. The plants were watered for 24 h before infection. The transformed Agrobacterium spp. with the target gene were activated until they reached an OD_600_ of approximately 1.2. The sample was resuspended in a 5% sucrose solution to adjust the OD_600_ value to 0.6–0.8. Subsequently, 100 μL of Silwet L-77 was added to 1 L of the 5% sucrose solution (this was done at the point of infection). All flower buds were immersed in the infection solution for 1–2 min. Subsequently, the immersed plants were kept in the dark for 1 day. Then, the plants were returned to normal growing conditions until seed maturity.

T1 (These represent the T0 generation transgenic seeds directly obtained following Agrobacterium-mediated transformation.) seeds were planted on 1/2 MS medium with kanamycin (50 µg/ml) to select transgenic plants. The stable transformation was confirmed in the T2 (These seeds are produced by self-fertilization of T1 plants.) and T3 (These seeds are derived from self-fertilization of confirmed homozygous T2 plants.) generations.

Subsequently, two independent homozygous lines (*KcRCB*-OE1 or OE1 and *KcRCB*-OE2 or OE2) with robust growth were selected for the study. The seedlings were cultivated in a controlled growth chamber at 21°C under a 16-h light/8-h dark photoperiod (light intensity: 150–200 μmol·m^-^²·s^-^¹) for four weeks before heat stress treatments. Four-week-old transgenic lines (OE1, OE2) and wild-type *Arabidopsis thaliana* (Col-0) were exposed to 45°C for heat stress in a constant-temperature incubator under dark conditions. The samples were harvested at 0, 5, 30, 120, and 240 min post-stress for quantifying the catalase (CAT) and peroxidase (POD) activities, as well as the relative electrical conductivity. We also estimated H_2_O_2_ levels and performed NBT staining of samples at 0 and 240 min to visualize accumulation of hydrogen peroxide and superoxide anions, respectively. Following sampling, heat treatment persisted until distinct phenotypic alterations (e.g., leaf wilting, chlorosis) were observed. Stressed plants were then transferred to a 21°C light incubator (16 h light/8 h dark cycle, 150 µmol m^-^² s^-^¹) for 3 days of recovery. Subsequent phenotypic assessments were performed after recovery using a kit manufactured by the Beijing Solepol Technology Company. We accurately measured 1 g of young green leaves and added 10 mL of distilled water. The mixture was then left to stand for 12 h and the conductivity was estimated as R1. The mixture was subsequently boiled for 30 min and then allowed to cool to room temperature and the conductivity was estimated as R2. The relative conductivity was then calculated according to the following formula: Relative conductivity = R1/R2. The data were analyzed using the Excel and Graphpad 9.0 software packages (Jambunathan, 2010). All kits for the determination of physiological and biochemical indicators were purchased from Solarbio Science & Technology Co., Ltd.

### 5.5 Analysis of high temperature tolerance in the *KcRCB-*overexpressing cotton plants

Cotton seeds were sterilized in histoculture flasks containing MS medium, followed by culturing in a constant temperature incubator set at 28°C in dark. This procedure was undertaken to obtain cotton stem segments. The Agrobacterium strain was used to infect the cotton stem segments. The segments were then cultured in the dark for three days. Thereafter, they were cultured under standard conditions to allow healing of wounds, root growth, and transplantation into soil after the growth of cotyledons for positive validation (Srivastava et al., 2023). The *KcRCB*-overexpressing transgenic and non-transgenic plants were cultivated at 28°C and then grown to 2–3 true leaves for high temperature tolerance analysis. The plants were treated at 45°C for 0, 5, 30, 120, and 240 min, respectively. Three biological replicates were set up for each treatment. Malondialdehyde (MDA) and proline (Pro) levels were evaluated at all time points using kits from the Beijing Solepol Science and Technology Company. The H_2_O_2_ and superoxide (NBT staining) levels were measured in the control and *KcRCB*-overexpressing plant leaves after 240 min of treatment. The data was analyzed and processed using the Excel and GraphPad 9.0 software.

### 5.6 Analysis of stomata in cotton leaves by blotting method

Stomata in the cotton leaves were observed using the nail polish and transparent tape blotting method (Yuan et al., 2020). A uniform layer of nail polish was applied to the vein-free area on the abaxial surface of cotton leaves and air-dried at room temperature for approximately 5–10 min. Then, a transparent tape with the adhesive side was pressed gently by fingers to fully contact the dried nail polish and peeled off to obtain the imprint, pasted onto a glass slide with the adhesive side, and observed under a light microscope at 40× magnification.

## Data Availability

The original contributions presented in the study are included in the article/**Supplementary Material**. Further inquiries can be directed to the corresponding author.

## Supplementary material

Supplementary Figure 1 The amino acid sequence of KcRCB.

Supplementary Figure 2 The conserved domain of RCB protein.

Supplementary File Amino acid information for RCB proteins from different plant species used for phylogenetic tree construction.
