# Supplementary figures and images for: Cloning and high temperature tolerance analysis of the thermal response related gene KcRCB in Karelinia caspia (Pall.) Less
Source: Front Plant Sci. 2025 Sep 17;16:1641916. doi: 10.3389/fpls.2025.1641916 (PMC12484188; doi:10.3389/fpls.2025.1641916)

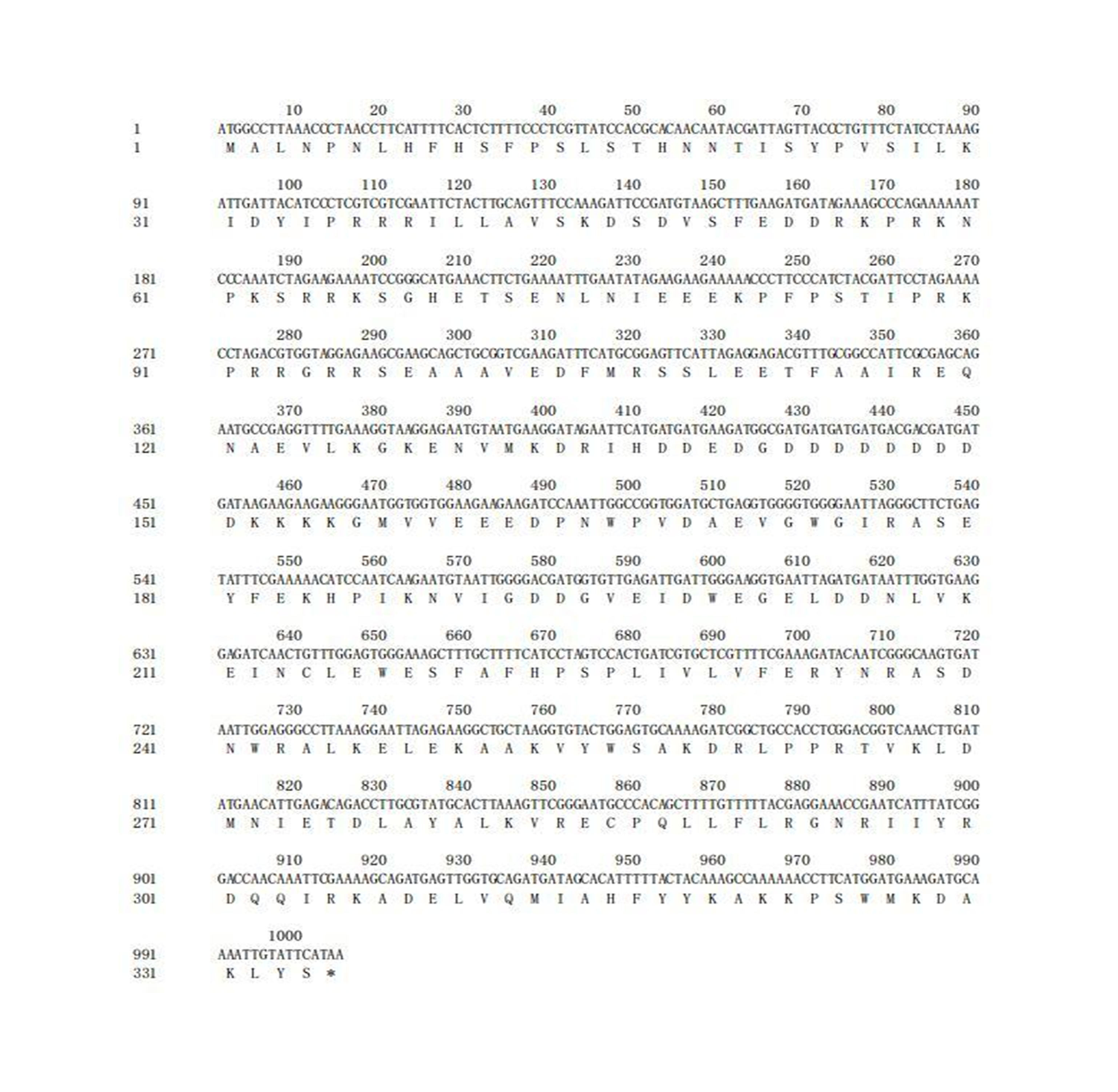

Supplement: Supplementary Figure 1 — The amino acid sequence of KcRCB. [file Image1.tif]

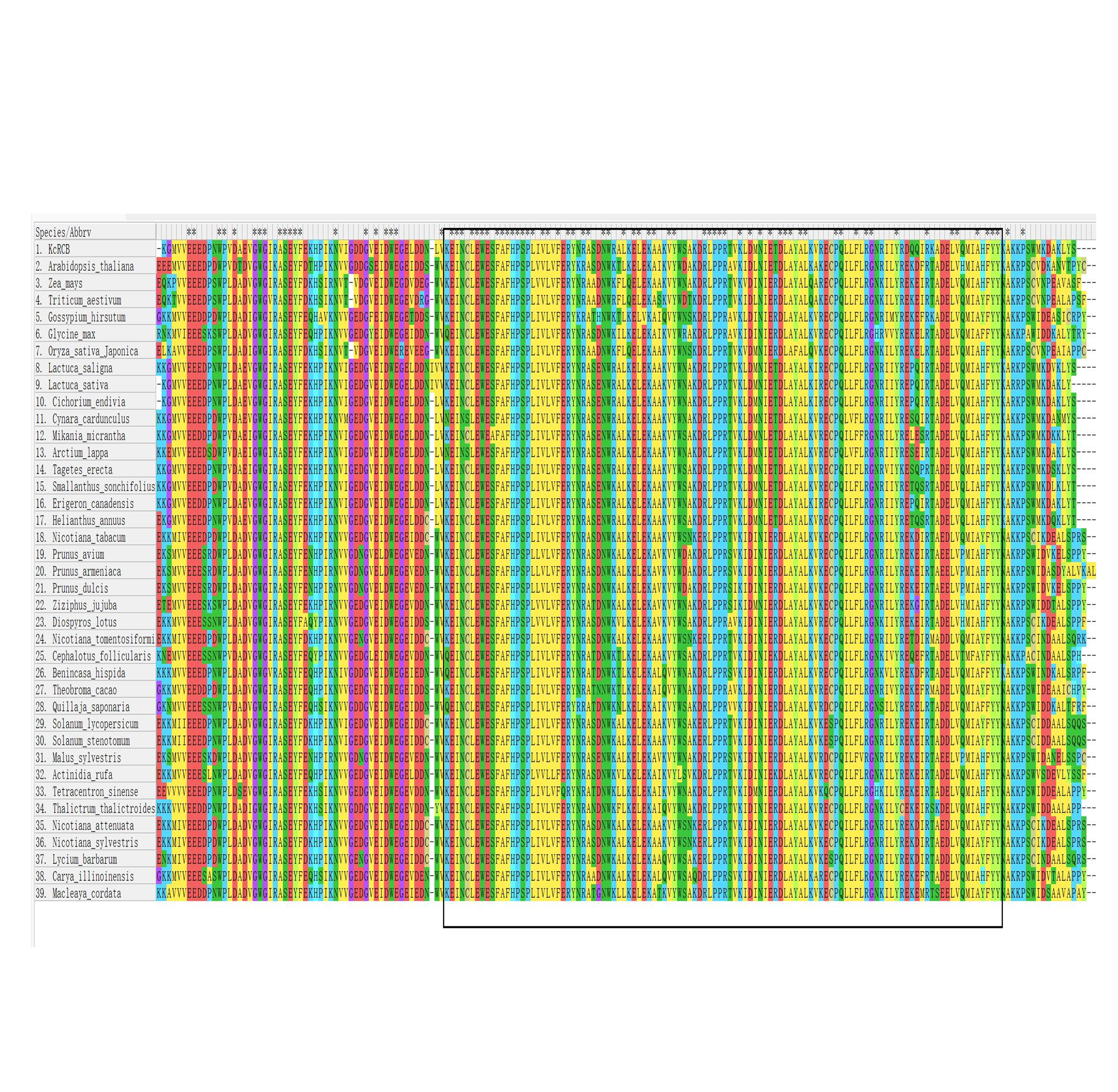

Supplement: Supplementary Figure 2 — The conserved domain of RCB protein. [file Image2.tif]
